# A Comprehensive Model for Estimating Heat Vulnerability of Young Athletes

**DOI:** 10.3390/ijerph17176156

**Published:** 2020-08-25

**Authors:** Wenwen Cheng, J. O. Spengler, Robert D. Brown

**Affiliations:** 1 College of Architecture, The University of Oklahoma, Norman, OK 73019, USA; 2 School of Public Health, Texas A&M University, College Station, TX 77843, USA; spengler@tamu.edu; 3 Department of Landscape Architecture and Urban Planning, Texas A&M University, College Station, TX 77843, USA; rbrown@arch.tamu.edu

**Keywords:** heat stress index, WBGT, young athletes thermal health, energy budget thermal model, ballfield design

## Abstract

Current methods for estimating heat vulnerability of young athletes use a heat index (HI) or a wet bulb globe thermometer (WBGT), neither of which fully include the environmental or physiological characteristics that can affect a person’s heat budget, particularly where activity occurs on a synthetic surface. This study analyzed and compared the standard methods, HI and WBGT, with a novel and more comprehensive method termed COMFA-Kid (CK) which is based on an energy budget model explicitly designed for youth. The COMFA model was presented at the same time to demonstrate the difference between a child and an adult during activity. Micrometeorological measurements were taken at a synthetic-surfaced football field during mid-day in hot environmental conditions. Standard methods (HI and WBGT) indicated that conditions on the field were relatively safe for youth to engage in activities related to football practice or games, whereas the CK method indicated that conditions were dangerously hot and could lead to exertional heat illness. Estimates using the CK method also indicated that coaches and staff standing on the sidelines, and parents sitting in the stands, would not only be safe from heat but would be thermally comfortable. The difference in thermal comfort experienced by coaches and staff off the field, versus that experienced by young players on the field, could affect decision making regarding the duration and intensity of practices and time in the game. The CK method, which is easy to use and available for modification for specific conditions, would lead to more accurate estimates of heat safety on outdoor synthetic surfaces in particular, and in sports with a high prevalence of heat illness such as football, and should be considered as a complementary or alternative preventive measure against heat.

## 1. Introduction

Exertional heat illness (EHI) is a major cause of death and disability among young athletes in the U.S. [1]. EHI is associated with symptoms such as persistent muscular cramps, anorexia, diarrhea, and a body-core temperature above 40 °C which will cause coordination difficulties, cognitive function reduction, and reduced endurance performance [2], and is the result of exercising in environmental conditions that are too hot. The highest incidence of EHI among participants of organized sport among all ages is in American football [3]. Additionally, as reported in the literature, there has been a considerable increase over the past three decades in EHI fatalities in football [3]. Among collegiate athletes, an analysis of NCAA Injury Surveillance Program data found that football comprised most (75%) EHI events and occurred at the highest rate during preseason practices [4]. In youth sport, the incidence of injury and death due to heat is also greatest in football. An analysis of data from the National High School Sports-Related Injury Surveillance System (2005/2006–2010/2011) found the exertional heat illness rate in football (4.42 per 100,000 athlete exposures) to be 11.4 times greater than that in all other sports in the high school dataset combined [2]. Further, it is estimated that between 2005 to 2009, more than 9000 high school athletes were treated for exertional heat illness annually [1]. The most time lost from athletics due to heat illness was among football players, and occurred most frequently in August during early season football practices [1]. In addition to illness, most heat-related deaths related to youth sport participation in the U.S. occur during participation in football [1,2].

In an effort to prevent heat-related illness and death in the broader context of outdoor activities and environments, and among young athletes in particular, a variety of metrics and indices have been developed and used in estimating environmental heat risks. The heat index (HI), as described by the National Oceanic and Atmospheric Administration’s National Weather Service [5], was initially created by Steadman [6] using ambient dry bulb temperature and relative humidity as input data:

(1) $$\begin{array}{cc} \mathrm{HI}=\;-42.379\; & +\;2.049\mathrm{Ta}\;+\;10.143\mathrm{RH}\;-\;0.225\mathrm{Ta}\times \mathrm{RH}\;-\;6.838\times 10^{-3}{\mathrm{Ta}}^{2}\\ & \;-\;5.482\times 10^{-2}R2\;+\;1.229\times 10^{-3}\mathrm{Ta}\times 2R\;\\ & \;+\;8.528\times 10^{-4}\mathrm{Ta}\times R^{2}\;-\;1.990\times 10^{-6}\mathrm{Ta}\times 2R2 \end{array}$$

where Ta is the ambient dry bulb temperature (°F), and RH is relative humidity (%).

This model derived a definitive scale of apparent temperature, which takes account of the effects of temperature and humidity on the reaction of humans. It has been widely used in studies to estimate urban environment heat exposure and to assess the risk of acquiring heat stress [7,8,9]. However, there are some limitations to the HI’s original development in several aspects when used for predicting young athletes’ heat stress during practice and games. For example, the microclimatic environmental variables such as sky view factor, ground surface temperature, surrounding surface temperature, and porosity of windbreaks were not considered in the model. Moreover, the mean radiant temperature that factors in short and longwave radiation, a key factor affecting human’s thermal comfort in hot conditions, was not considered. Further, the human parameters were designed and assumed for an adult with regular clothing and a moderate working load (the basic human body dimensions were designed for a typical adult human with a height of 1.7 m and a weight of 67 kg; the heat production is for a person walking outdoors at a normal speed, 1.4 m/s, the clothing resistance was for a shirt and long pants). These missing considerations limit the applicability of HI for predicting heat stress levels under a microclimatic environment for a young person engaged in physically demanding activities. The effects of radiation heat obtained from the sun and surrounding environment, for example, are the most important factor in determining the outdoor human energy budget in hot weather. Additionally, human body dimensions, especially body surface area to mass ratio, are much higher for young people than adults, which results in additional heat exchange with the environment. Moreover, heat production for intensive activity is much higher than walking at a moderate speed. For example, the heat production for a 10-year-old boy playing football can be almost double the heat production of an adult man walking at a speed of 2.5–3.0 mph (408 to 210 W/m^2^). In addition, clothing resistance would greatly affect the thermal comfort level, especially for athletes [10].

The wet bulb globe temperature (WBGT) is another climatic index used worldwide that was first developed to prevent heat illness in military training camps and then used as a standard by ISO 7243 [11]. It is also often used as a preventive measure against heat in sanctioned high school athletic events. The WBGT is calculated using the following formula:

(2) WBGT=0.7Tw+−0.2Tg+0.1Ta

where Ta is the air temperature (°C), Tg is black globe temperature measured by a globe 15 cm in diameter (°C), and Tw is the natural wet bulb temperature (°C).

If the WBGT index is above 28 °C (82 °F), consideration should be given to canceling or rescheduling ongoing competitive athletic events until conditions giving rise to heat stress are lessened [11]. However, some studies have concluded several limitations of using the WBGT index to predict young athletes’ thermal stress conditions. Limitations include a failure of the index to include microclimatic factors such as sky view factor, ground surface temperature, surrounding surface temperature, or windbreak porosity [12]. For human factors, the interpretation of the thermal sensation of the model was originally interpreted by workers and managers working in the field, which cannot represent the athletes’ thermal sensations [13]. In addition, the WBGT values do not vary linearly as a function of the metabolic rate (M) [14]. Using the average value of different M values is not appropriate and can lead to detrimental health outcomes, especially for athletes involved in intensive athletic activities such as football. Moreover, although different clothing conditions other than “normal working clothes” (with a clothing resistance of 0.6) were proposed to be added into the model, the clothing factor is independent of the climate and the WBGT values [14]. However, the clothing factor can be strongly affected by climate according to a previous study, especially for athletes [10].

The most important limitation of the WBGT application is its shape. People, and young athletes in particular, are not spherical in shape, but rather are more cylindrical. Results obtained from a spherical instrument are not appropriate to be applied to a cylindrical human form. A recent study [15] identified the magnitude of this error and suggested the use of a cylindrical instrument (or cylindrical model) to eliminate that error. When applying the WBGT, the Occupational Safety and Health Administration provides two options for the input of climate data: using the WBGT meter; or calculating a value using local weather station data. However, calculation of WBGT from weather station data does not include any terrestrial radiation, which could introduce a large error when compared to field measurements.

A recently developed model, however, has the potential to mitigate the errors and limitations of HI and WBGT. The COMFA-Kids (CK) model was developed based on the COMFA model [16]. It is the first energy budget model to predict children’s outdoor thermal comfort level considering both microclimatic and human parameters [17]. The basic equation of the CK model is

Energy Budget = M + Rabs − Conv − Evap − Tremitted(3)

where M is the metabolic energy for heating up the body (W/m^2^), Rabs is the absorbed solar and terrestrial radiation (W/m^2^), Conv is the sensible convective heat exchange (W/m^2^), Evap is the evaporative heat loss (W/m^2^), and TRemitted is the emitted terrestrial radiation (W/m^2^).

The CK model has several characteristics that make it potentially a valuable method for estimating young athletes’ heat stress levels:

(a) It is a comprehensive model that includes all the climate, microclimatic, and human parameters affecting human energy budgets;

(b) It was developed based on children’s thermal exchange characteristics, taking their higher metabolic rate, higher skin temperature, higher surface area-to-mass ratio, and lower sweating rate into consideration;

(c) It is an open-architecture model that allows investigation of each stream of energy independently and can identify site characteristics that need to be modified to provide thermally safe conditions. For example, microclimatic environmental data such as sky view factor and albedo (reflected light or radiation) of the ground can be set based on the real conditions, while variables such as clothing resistance and metabolic heat production for different activities can be specifically input and tested.

This study used HI, WBGT, and the CK model to analyze and predict the level of heat stress that would be encountered by young athletes playing football during two hot days and compared the results. The COMFA model was used to demonstrate the energy budget value for an adult.

## 2. Materials and Methods

### 2.1. Microclimatic Data Collection

To calculate the HI, WBGT, and the energy budget values for a young athlete and an adult coach using the COMFA model and the CK model, microclimatic data were collected on an artificial turf football field at Veteran’s Park, College Station, Texas on 10 October 2019 from 1:30–2:30 p.m., and 19 May 2020 from 1:30–3:30 p.m.

A portable weather station (MaxiMet GMX501, Gill Instruments, Hampshire, UK) collected a full suite of microclimatic variables, including air temperature (Ta), direct solar radiation (SR), wind speed (Ws), and relative humidity (RH). This weather station was mounted 1.5 m off the ground. A Campbell Scientific Black Globe Thermometer (BLACKGLOBE_L, Campbell Scientific, Logan, UT, USA) was used to measure the black globe temperature. A thermal camera FLIR E5 (FLIR System, Inc., Wilsonville, OR, USA) measured the surface temperature of the field and the environment. Data from MaxiMet GMX501 were collected with a CR310 data logger (Campbell Scientific, Logan, UT, USA) at 10-s intervals. Data from all other instruments were collected with a CR3000 data logger (Campbell Scientific, Logan, UT, USA) at the same time intervals.

The Kestrel WBGT Heat Stress Tracker and Weather Meter was designed to detect microclimatic data and heat-related indices, including WBGT, Thermal Work Limit (TWL), and HI for outdoor workers and athletes. The Kestrel 5400 Heat Stress Tracker with LiNK (Kestrel Instruments, Boothwyn, PA, USA) was used in this study on 19 May 2020, to obtain the WBGT and HI values directly. Data were stored every 10 s. Kestrel LiNK (Kestrel Instruments, Boothwyn, PA, USA) for Windows was used to export the data.

### 2.2. Calculate Heat Stress Level Using HI, WBGT, the CK, and COMFA Model

The HI and WBGT heat stress values were calculated using Ta and RH data from Maximet. Tg was measured by BLACKGLOBE_L. Tw was calculated using RH and Ta based on Stull’s [18] equation that has been validated and widely used:

(4) $$\begin{array}{cc} T_{w}=T_{a}\;a\tan & \left[0.151977\;{\left(RH\%+8.313659\right)}^{\frac{1}{2}}\right]\\ & \;\;\;\;\;+a\tan\left(T_{a}+RH\%\right)\\ & \;\;\;\;\;-a\tan\left(RH\%-1.676331\right)\\ & \;\;\;\;\;+0.00391838{\left(RH\%\right)}^{\frac{3}{2}}a\tan\left(0.023101RH\%\right)-4.686035 \end{array}$$

The HI and WBGT heat stress values can also be obtained from the Kestrel Heat Stress Tracker.

Two energy budget models, the CK model, and the original COMFA model were used to determine the energy budget level for a 10-year-old boy playing football on the field (RMR = 52 W/m^2^), and a 40-year-old man coaching on the sidelines (RMR = 42 W/m^2^). The MET rate is 7 for the boy who is “running moderately in a football game” and 4 for the man who is “coaching football” based on the 2011 Compendium of Physical Activities [19]. WHO standards for heights and weights were used to calculate other physiological values in the model.

The CK model yields an energy budget value with the unit of W/m^2^, while HI and WBGT result in the units of °C. To compare these, Harlan et al.’s [7] relationship between the energy budget values and HI values was used in this study (EB = 60–120 W/m^2^, HI = 26.7–31.7 °C, Label = Caution; EB = 121–200 W/m^2^, HI = 32.2–40 °C, Label = Extreme caution; EB = 201–339 W/m^2^, HI = 40.6–53.9 °C, Label = Danger; EB = 340 or higher W/m^2^, HI = 54.4 °C or higher, Label = Extreme danger). Table 1 shows each heat stress category of the HI, WBGT, and the COMFA model for athletic activities (we use the same category for the CK model).

## 3. Results

### 3.1. Microclimatic Conditions and Heat Stress Level Using HI, WBGT, the CK and COMFA Model of Testing Days

Table 2 shows the weather conditions and heat stress values of the two test periods of 1:30–2:30 p.m. on 10 October 2019 and 1:30–3:30 p.m. on 19 May 2020. Air temperature (Ta) was similar between the two test times (mean Ta is 34.0 °C on 10 October and 33.3 °C on 19 May). The date 19 May was more humid and less windy (mean RH was 48% and mean Ws was 2.4 m/s) than 10 October (mean RH was 42.9% and mean Ws was 4.04 m/s). Direct SR was much higher on 19 May (mean SR was 886.8 W/m^2^) than 10 October (mean SR was 574.3 W/m^2^). Mean HI (36.2 °C) and WBGT (28.2 °C and 28.8 °C) of the two test periods were similar, while EB values for a 10-year-old young athlete and a 40-year-old coach on 19 May (376.5 W/m^2^ and 228.3 W/m^2^) were much higher than those of 10 October (254.6 W/m^2^ and 108.2 W/m^2^).

### 3.2. Comparison of Heat Stress Level Using HI, WBGT, the CK and COMFA Model

**Figure 1:**
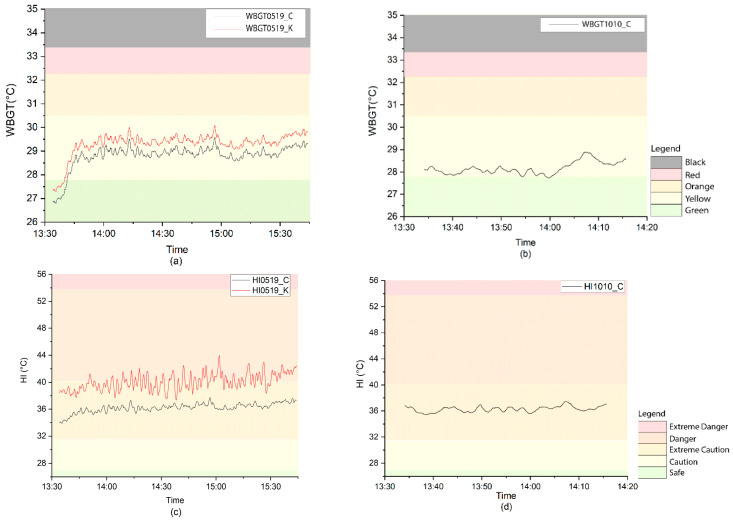
(**a**) Calculated WBGT heat stress and Kestrel WBGT on 19 May 2020; (**b**) calculated WBGT on 10 October 2019; (**c**) calculated HI heat stress and Kestrel HI on 19 May 2020; (**d**) calculated HI on 10 October 2019.

Figure 1a,c show the HI and WBGT values calculated from microclimatic data using Maximet and direct from Kestrel on 19 May 2020; Figure 1b,d show the HI and WBGT values on 10 October 2019. All the calculated HI values were under the “Extreme Caution” level of the two days. Kestrel’s HI values were higher than the calculated values. More than half of the Kestrel HI values were under “Danger”, and the rest were under “Extreme Caution”.

For calculated WBGT values on the two days, based on the regional heat safety thresholds for athletes in the U.S. [18], the activity guidelines for the two periods were from “Green—normal activities” to “Yellow—use discretion for intense or prolonged exercise”. Kestrel’s WBGT values were slightly higher than the calculated values while at the same activity guidance category. The calculated WBGT and HI values and Kestrel’s values showed a strong relationship (R^2^ = 1). The higher Kestrel HI and WBGT values were significantly higher because of the higher Ta, RH, and Tw value measurement by Kestrel which was likely due to a radiation error.

**Figure 2:**
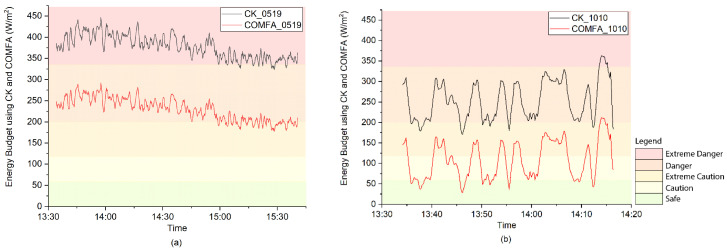
Energy budget values and heat stress levels of the young athlete and the adult coach on (**a**) 19 May 2020 and (**b**) 10 October 2019.

Figure 2 shows the EB values of a 10-year-old boy playing football and a 40-year-old man coaching football on the two test days. Based on the EB threshold for heat stress, on 19 May, during most of the test time, the young football player was under “Extreme Danger”, while the adult coach was under the “Caution” and “Extreme Caution” levels. On 10 October, most of the EB young athlete values were in “Danger”, while most of the coach’s EB values were at “Caution” or “Safe”.

Table 3 shows the heat stress level for the two test days of the HI, WBGT, the CK model, and the COMFA model using the Maximet data. We considered the five heat stress levels of WBGT (Green, Yellow, Orange, Red, and Black) the same function as the five levels of HI (Safe, Caution, Extreme Caution, Danger, and Extreme Danger) to make the results comparable.

Using the microclimatic data collected by the same device Maximet, the heat stress level calculated by the CK model for a 10-year old boy playing football was from danger to extreme danger, while HI and WBGT were from safe to extreme caution. When comparing adults’ level by the COMFA model, children’s heat stress level was one–two levels higher than adults’.

**Figure 3:**
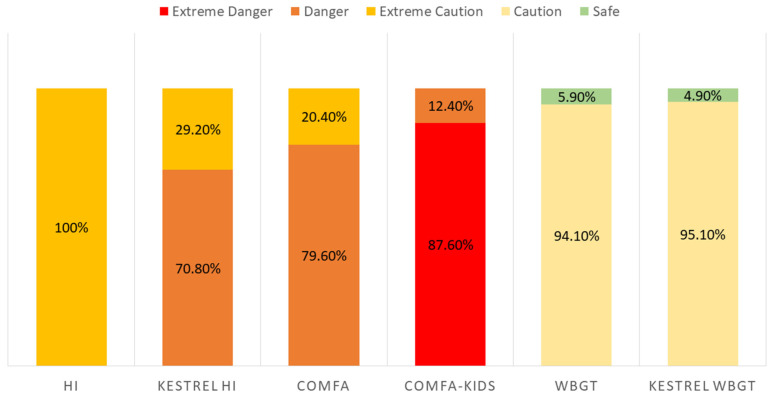
Percentage of time under each heat stress level on 19 May 2020, using HI, WBGT, the COMFA model, and the CK model.

Figure 3 shows the percentage of time under each heat stress level on 19 May 2020, using HI, WBGT, the COMFA model, and the CK model by different devices, Maximet and Kestrel.

Compared with the heat stress level from the COMFA model and the CK model, only the Kestrel HI was similar to the adult’s result (the overlap percentage is 91.2%), while all the other measurements underestimated the heat stress level of the young athlete and the adult. As stated above, Ta and RH values from the Kestrel were higher than data from Maximet. When using the Ta and RH value from Kestrel instead of Maximet as input data for the COMFA model, the agreement of the results from the Kestrel HI and the COMFA model dropped from 91.2% to 21.3%. The Kestrel HI still underestimated the COMFA results when using the same dataset.

### 3.3. Comparison of Heat Stress Level of a Standing Coach, a Sitting Parent, and a Young Football Player

**Figure 4:**
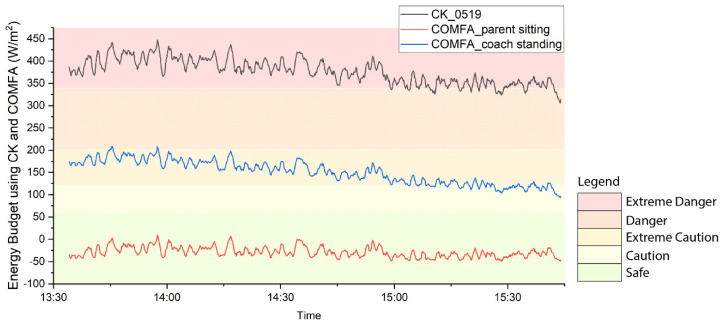
Heat stress level of a young athlete (10 year-old boy) playing football, an adult coach (a 40-year-old man) standing outside the field, and an adult parent (a 40-year-old man) sitting under an oak tree.

The coach may stand off the artificial turf football field on real grass which has a much lower surface temperature than artificial turf, and the parents may sit outside the field under some shade. Figure 4 shows the energy budget of: a 10-year-old boy playing football in an artificial turf field, a 40-year-old adult coach standing outside the field with real grass as the ground material (surface temperature is similar to air temperature), and a 40-year-old adult parent sitting under an oak tree (transmissivity is 17.5%, sky view factor is 50%). The coach is under “Extreme Caution” and may not understand the danger to the players, and the parent is even more comfortable and not having the same thermal sensation with his child, while the boy athlete is experiencing an “Extreme Danger” level of heat stress.

## 4. Discussion

HI and WBGT are the heat stress indices frequently used in predicting or assessing young athletes’ heat stress during training or competition. However, previous studies have identified limitations of applying these indices, including: they are empirical relationships that might not apply in all climate zones [12]; they either do not consider the shape of a person (HI) or they use a globe-shaped instrument which introduces potentially very large errors into the measurements [13,14]; they were both developed for adults and do not consider the characteristics of children, and they are essentially closed systems that do not identify what part of the environment is problematic and do not allow for remediation [12]. In contrast, the COMFA and CK models are validated and comprehensive energy budget models that predict humans’ outdoor thermal comfort levels by consideration of both microclimatic and human parameters, providing a more complete explanation of the energy exchange between the human body and the environment. Table 4 demonstrates the environmental and human parameters included in HI, WBGT, the COMFA model, and the CK model.

When using HI, WBGT, the CK model, and the COMFA model to present the heat stress level for a 10-year-old boy playing a football game and a 40-year-old adult coaching football on the sidelines, large discrepancies of heat stress levels were obtained from the results using different heat stress models/indices. On 19 May 2020 noontime, a young athlete playing football would experience “Extremely Dangerous” conditions according to the CK model, while the coach would be under the “Extreme Caution” to “Danger” category using the COMFA model. HI estimated “Extreme Caution” to “Danger”, while WBGT assessed the conditions as “Green” (normal activities) to “Yellow” level (use discretion for intense or prolonged exercise). During 10 October noontime, the young athlete’s heat stress level was under “Extreme Caution” to “Danger”, the adult coach was under “Safe” to “Caution”, HI level was “Extreme Caution”, while WBGT level was “Green” to “Yellow”.

Our study found major differences among the heat stress results using different methods/indices. We found that HI and WBGT both underestimate the heat stress level for both the young athlete and adult coach. The reason is that neither HI nor WBGT are comprehensive models able to predict heat stress as they do not analyze the full suite of climate factors’, microclimatic environmental factors’, and human parameters’ effects on the energy exchange between the human body and the environment. Additionally, both HI and WBGT were developed based on an adult man with normal physical activities and wearing normal working clothes that are not appropriate for athletes.

This study also found that a young athlete’s heat stress level would be higher than the adult coach. This could be due to an underestimation of heat stress in the young athlete using HI and WBGT. The physiological differences between youth and adults are accounted for in the CK model, but not in HI or WBGT, resulting in different thermal regulation calculations. Children, for example, have higher resting metabolic heat production than adults [22], lower sweating rate (half capacity) [23], and higher convection heat exchange due to their higher surface area to mass ratio [24]. Additionally, the MET rate for a football player and a coach is different (7 and 4) which will cause a huge difference in the metabolic heat production in the player’s and coach’s body which is not considered or changeable in HI and WBGT.

This study also demonstrates the different HI and WBGT results using different devices, Kestrel and the Maximet GMX 501 weather station. As d’Ambrosio Alfano et al. [14] mentioned, many instruments are, in some cases, impossible to calibrate and not using standard sensors can lead to large errors.

The results of this study suggest the need for greater specificity in measurement as a preventive measure against heat in youth sport. This is particularly important given the threat that climate change poses to youth sports such as football, and outdoor play. Heat is identified as the deadliest hazard in the developed world, and global warming, found to be increasingly severe, brings intense heat with the associated risks of heatstroke, hyperthermia, and other health issues [25]. It is also important given findings that indicate synthetic surfaces are much hotter than natural surfaces [26], but this is not captured with measures such as HI. The authors recommend that the CK measure be given consideration by organizations and associations dedicated to sport science, athletic training, and coaching to determine its feasibility as a complementary or alternative preventive measure against heat. Potential advantages to using CK would be to help coaches understand the difference in heat exposure from the players moving around on the field to the coaches and others standing on the sideline and in the stands, the severity and danger of heat on synthetic surfaces as measured by CK, and the potential need to adjust safety protocols, particularly for youth football, based on measures of heat using CK. Additionally, further research is recommended to test the CK tool in a variety of environmental conditions, and further develop the technology to allow for ease of use through an app or device that could be integrated into the play environment.

## 5. Conclusions

This study used HI, WBGT, the COMFA model and the CK model to estimate the heat stress level of a 10-year-old boy playing football, a 40-year-old male coach coaching, and a 40-year-old male parent sitting in the stands on an early summer day and an early autumn day. The results showed that HI and WBGT underestimated the heat stress level for both the adults and the young boy. Also, a young athlete’s heat stress level would be higher than the adult coach and the parent. The CK model, an open-architect model that considers both environmental and human parameters, can provide those responsible for the safety of youth sport participants with more accurate estimates of heat stress levels. The model can be used to inform policy and decision making for outdoor youth sport practices and games during hot weather.

## Figures and Tables

**Table 1:** HI, WBGT and the COMFA model heat stress category.

Category	HI	COMFA	Category *	WBGT
Safe	<26.7 °C	<60 W/m^2^	Green	<27.8 °C
Caution	26.7–31.7 °C	60–120 W/m^2^	Yellow	27.9–30.5 °C
Extreme Caution	32.2–40 °C	121–200 W/m^2^	Orange	30.6–32.3 °C
Danger	40.6–53.9 °C	201–339 W/m^2^	Red	32.2–33.3 °C
Extreme Danger	>54.4 °C	>340 W/m^2^	Black	>33.4 °C

* Value of WBGT for athletic activity is from Cat 3 where the measurements were taken. The heat safety categories for athletics are: Green: normal activities—provide at least three separate rest breaks each hour with a minimum duration of 3 min each during the workout. Yellow: use discretion for intense or prolonged exercise; provide at least three separate rest breaks each hour with a minimum duration of 4 min each. Orange: maximum practice time is 2 h. Red: maximum practice time is 1 h. Black: no outdoor workouts. Delay practice until a cooler WBGT is reached. See details at: https://www.weather.gov/rah/WBGT [20], and Grundstein et al. [21].

**Table 2:** Meteorological data and heat stress/energy budget values from HI, WBGT, the COMFA model, and the CK model.

Test Time		Ta (°C)	RH (%)	Ws (m/s)	SR (W/m^2^)	EB Athlete (W/m^2^)	EB Coach (W/m^2^)	HI (°C)	WBGT (°C)
October	Max	35	46	7.14	950	383.7	232.1	37.6	28.9
	Min	33.3	41	0.98	193	147.2	9.2	35.2	27.6
	Mean	34.0	42.9	4.04	574.3	254.6	108.2	36.2	28.2
May	Max	34.2	56	5.8	1005	468.1	308.4	38.3	29.8
	Min	31	44	0.5	743	284.5	144.4	33.7	26.6
	Mean	33.3	48	2.4	886.8	376.5	228.3	36.2	28.8

**Table 3:** Heat stress level using HI, WBFT, the CK model, and the COMFA model.

Index/Model	Heat Stress Level	
	19 May	10 October
WBGT	Safe-Caution	Caution
HI	Extreme Caution	Extreme Caution
CK	Danger-Extreme Danger	Extreme Caution-Extreme Danger
COMFA	Extreme Caution-Danger	Safe-Danger

**Table 4:** Environmental and human parameters in HI, WBGT, the COMFA model, and the CK model.

Model	Environmental Parameters								Human Parameters	
	SR	Ta	RH	Ws	SVF	TR	DR	RR	M	Cl
HI		✓	✓							
WBGT	✓ ^1^	✓	✓	✓ ^1^					✓ ^2^	
COMFA	✓	✓	✓	✓	✓	✓	✓	✓	✓	✓
CK	✓	✓	✓	✓	✓	✓	✓	✓	✓ ^3^	✓

Legend: SR: solar radiation; Ta: air temperature; RH: relative humidity; Ws: wind speed; SVF: sky view factor; TR: terrestrial radiation; DR: diffused radiation; RR: reflected radiation from the environment (building and ground); M: metabolic rate; Cl: clothing condition. ^1^ There is no direct input of SR, RH, and Ws for WBGT, while the globe temperature is influenced by wind speed and solar irradiation, and the wet temperature is affected by relative humidity. ^2^ WBGT varies according to different metabolic activities [11]. ^3^ Metabolic rate of children was used in CK.
